# Comparison of Bi- and Tri-Linear PLS Models for Variable Selection in Metabolomic Time-Series Experiments

**DOI:** 10.3390/metabo9050092

**Published:** 2019-05-09

**Authors:** Qian Gao, Lars O. Dragsted, Timothy Ebbels

**Affiliations:** 1 Department of Nutrition, Exercise and Sports, University of Copenhagen, 1958 Frederiksberg, Denmark; qian@nexs.ku.dk (Q.G.); ldra@nexs.ku.dk (L.O.D.); 2 Computational and Systems Medicine, Department of Surgery and Cancer, Imperial College London, London SW7 2AZ, UK

**Keywords:** time series, PLS, NPLS, variable selection, bootstrapped-VIP

## Abstract

Metabolomic studies with a time-series design are widely used for discovery and validation of biomarkers. In such studies, changes of metabolic profiles over time under different conditions (e.g., control and intervention) are compared, and metabolites responding differently between the conditions are identified as putative biomarkers. To incorporate time-series information into the variable (biomarker) selection in partial least squares regression (PLS) models, we created PLS models with different combinations of bilinear/trilinear **X** and group/time response dummy **Y**. In total, five PLS models were evaluated on two real datasets, and also on simulated datasets with varying characteristics (number of subjects, number of variables, inter-individual variability, intra-individual variability and number of time points). Variables showing specific temporal patterns observed visually and determined statistically were labelled as discriminating variables. Bootstrapped-VIP scores were calculated for variable selection and the variable selection performance of five PLS models were assessed based on their capacity to correctly select the discriminating variables. The results showed that the bilinear PLS model with group × time response as dummy **Y** provided the highest recall (true positive rate) of 83–95% with high precision, independent of most characteristics of the datasets. Trilinear PLS models tend to select a small number of variables with high precision but relatively high false negative rate (lower power). They are also less affected by the noise compared to bilinear PLS models. In datasets with high inter-individual variability, bilinear PLS models tend to provide higher recall while trilinear models tend to provide higher precision. Overall, we recommend bilinear PLS with group x time response **Y** for variable selection applications in metabolomics intervention time series studies.

## 1. Introduction

Metabolomics is a widely applied technology for capturing the perturbations of metabolites in biological systems and for discovery of dietary and health biomarkers. Liquid chromatography–mass spectrometry (LC-MS), nuclear magnetic resonance spectroscopy (NMR), and gas chromatography–mass spectrometry (GC-MS) are most commonly employed in metabolomics studies providing information-rich, high throughput data [1]. Such data contains information on hundreds or even thousands of metabolites, resulting in challenges for both data pre-processing and statistical analysis [2].

Biomarker discovery in metabolomic studies consists of several stages: collection of biological samples under different conditions; application of analytical techniques for characterising the “unknown” metabolome; extraction of information from raw analytical data; statistical analysis to select putative biomarkers with the capacity to discriminate the samples from different conditions; and further studies to validate the performance of selected biomarkers [3]. Selection of variables (putative biomarkers) plays an important role in the process as it determines the scale and outcome of later validation studies [4]. It is crucial to keep the number of selected variables at a reasonable level without compromising the number of true positives.

**Figure 1:**
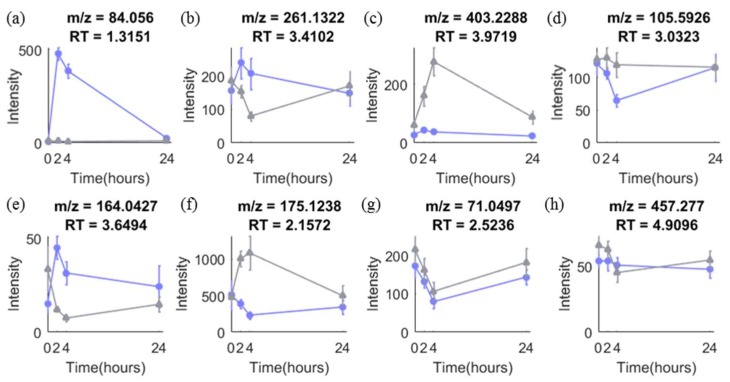
Typical temporal profiles of metabolites observed in metabolomics data from our onion study with a time-series design. (**a**)–(**h**) are temporal profiles of eight metabolites in control (grey) and intervention (purple) group. More details are explained in Text S1.

Time-series design has been adopted in many metabolomic studies for both biomarker discovery and validation stages. It is advantageous because it allows discovery of biomarkers responding to an intervention and provides time response information of biomarkers, which is of importance to select the best time window for sampling [5]. Figure 1 shows eight different types of temporal profiles typically seen in response to intervention in acute metabolomic studies (<24 h). Metabolites responding differently between the groups in such studies may vary in their temporal response profiles as seen in (**a**)–(**f**). Other metabolites (**g**)–(**h**) show no difference in response between control and intervention, or vary randomly over time, which is often the case for the majority of metabolites.

A time-series design yields more information but also leads to more complex data. Not only are the variables correlated but also temporal autocorrelation exists between time points. The classical supervised multivariate approach adopted in many metabolomic studies is PLSDA followed by variable (biomarker) selection [6]. PLSDA is a classification method based on classical PLS regression where the response variable, **y**, is categorical and represents which treatment group each sample belongs to [7]. Normally the model is built on the data acquired from a single time point, from combined time points or on pooled samples. However, in this case, only treatment group information is used while all the time response information is ignored.

Some attempts have been made to take time-series information into account during PLS modelling. One approach is to use time of sampling or maturity of the process as the response variable, **y**, [8] which has been applied in a small number of metabolomic studies [9,10]. The problem with this method is that it works well only when there is a linear relation between variables and time, which is often not the case (see Figure 1). Another approach is piecewise Orthogonal Projections to Latent Structures (OPLS), which uses a set of sub-models to describe the changes between successive time points [11]. This does not assume any linear trend between data and time, which makes it suitable for the analysis of non-linear response over time. However, the time-series information is distributed in a range of sub-models which hinders interpretation. A variety of non-PLS methods could also be adopted for modelling metabolomics time-series data but there are some limitations. Autoregressive moving average with exogenous inputs models (ARMAX) or space-state models can be used to describe the temporal profiles, but typically requires more time points (>10) [12]. Smoothness or its combination with dimension reduction method have also been developed and applied for time-series data [13]. However, all the methods above mainly focus on predicting response to a treatment over time instead of selecting important variables which discriminate between different treatments. More investigation of time-series models using PLS for metabolomics analyses is therefore needed, especially with respect to variable selection, in order to provide better guidance on optimal data analysis of such datasets. Conventionally, metabolomic time series data are constructed into a two-way structure (Sample × Metabolite) in PLS modelling where the time response information is overlooked. To incorporate such information into the data structure, time can be considered as the third mode. In this study, five different bi- and tri-linear PLS models were used to identify important variables contributing to the difference between groups in intervention response metabolomics studies with a time-series design. The variable selection performance of the five models were evaluated on both simulated and real datasets to provide insight into the most appropriate modelling approach for intervention response time series experiments.

## 2. Materials and Methods 

### 2.1. PLS and NPLS

PLS is a latent variable based multivariate linear regression between predictor variables (**X**) and dependent variables (**Y**), which aims at maximizing the covariance between the **X** and response **Y** [14]. N-PLS is an extension of PLS to multiway data [15] where **X** is an array with more than two dimensions (also referred to as ways or modes). Compared to PLS, N-PLS provides simpler models with relatively few parameters and avoids the interference between information from different modes [16]. Metabolomic data with a time-series design can be structured as a two-way table of dimensions I×J, where I=S×T (*S* = number of subjects; *T* = number of time points), J= number of metabolites. During the analysis, such data is divided into subsets according to time points for further analysis separately or analysed as a whole. In either case, the time-series information is not used by the model and correlation between samples collected at different time points from the same subject is lost. To make use of this autocorrelation between samples, we transformed the metabolomics time-series data into a three-way array with a size of S×J×T and analysed it using N-PLS. In the current paper, both two-way PLS and N-PLS models are used to analyse metabolomic time-series data and they are referred to as bi-PLS (bilinear-PLS) and tri-PLS (trilinear-PLS) models, respectively. 

### 2.2. PLS-DA and Dummy **Y**

In standard PLSDA, class labels indicating the group membership of each sample are used as dependant **Y** (dummy **Y**), e.g., y for the intervention group sample is 1 and for the control group sample is 0 or −1. However, samples obtained from different time points can be very different within the same class causing large variation within classes and consequently lead to poor predictions. Dividing the data and building separate models for each time point can reduce this problem but in this case, there are fewer observations for each comparison, and most importantly, the time response information is not modelled. To include such variation into the dependent **Y** and provide more guidance on the separation of samples, we created a new ‘time response’ dummy **Y** to reflect how the metabolites respond to the intervention with time. Specifically, for the target metabolites (Figure 1a–f), their excretion experiences an increase and a decrease within a certain time frame i.e., their intensities are higher or lower in the middle of the time-series than that at the beginning or at the end of the time-series. Therefore, we assign the samples acquired from the middle of the time-series capturing the high intensities of the target metabolites to a ‘response class’ and samples acquired from the first and last time point to a ‘no-response class’. Samples from these two classes are labelled with 10 and 1 respectively, which would be subsequently used as dummy **Y** in further PLSDA modelling. In our experiments, the model performance improved with increasing magnitude of the ‘time response class’ until it reached around 10. Therefore, 10 was used as the label for ‘response class’ in this paper. The value of the time response should be a user-defined value and it can be adjusted by testing with a range of values to find out the optimal one achieving the best predictive performance (Q^2^ or area under the ROC curve) of the model. 

### 2.3. Comparison of Variable Selection by Five PLS Models 

**Figure 2:**
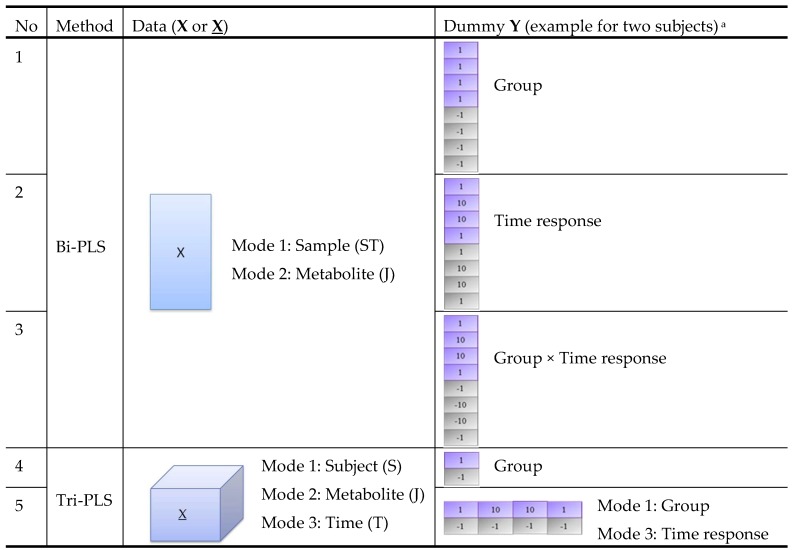
Structure of five PLS models for comparison. ^a^ The dummy **Y** in this figure is an example for data obtained from eight samples collected from two subjects at four time points (0, 2, 4, 24 h after intervention). Dummy **Y** in purple and grey colour corresponds to samples collected from Subject 1 (from intervention group) and Subject 2 (from the control group), respectively.

In order to take advantage of the time-series data structure and to make use of both group and time response information, we combined different PLS models (bi-PLS or tri-PLS) with different dummy **Y**s (group or time response labels) as shown in Figure 2. Models 1–3 are bi-PLS models built on a two-way matrix **X** of size ST×J. Model 4–5 are tri-PLS models built on a three-way array **X** of size S×J×T. For models 1–4, group labels, time response labels or their products are used as a one-way dummy **Y**. Model 5 uses a two-way dummy **Y** with group label as the first mode and time response label as the second mode. We note that model 1 only addresses group differences, while model 2 only addresses time response changes. Since we are interested in both group and time responses, we included these two basic models against which to compare the more complex models 3–5. The five PLS models were applied on the same datasets and their performances were compared.

The focus of this paper is on the ability of the models to highlight variables important to the time-treatment response. In PLS regression, variable selection is used to improve model performance to provide better predictions [17]. It identifies variables with large influence on the model, which could be used to interpret the model and to be investigated as potential biomarkers in further studies. In the current paper, VIP scores were calculated to identify the relevant variables and a bootstrap procedure was adopted to estimate VIP uncertainty.

### 2.4. Datasets

#### 2.4.1. Simulated Datasets

In order to assess the variable selection performance of the five PLS models, a data simulation procedure is proposed to simulate the time-series metabolomic dataset. For a simulated dataset, we generated J variables and for each variable j, the observations for a subject *s* in group *g* are generated according to the following equation:

$$x_{sg}=\;\mu_{g}\circ \left(b_{s}+w_{s}+\varepsilon_{s}\right)$$

where ∘ denotes the entry-wise product and $\mu_{g}=c+at^{\alpha }e^{-\beta t}$. $\mu_{g}$ is the vector containing the values of the mean curve for the group g, of dimension 1×T and t is the time. c, a, α, β are generated from uniform distributions, the intervals of which are adjusted to create different temporal profiles, as shown in Figure 1a–h for both intervention and control groups (see Figure S1). The 1 × T vector $b_{s}$ controls inter-individual variability, which follows a normal distribution with zero mean and covariance matrix $\sigma_{b}^{2}1_{T}$, where **1** denotes a matrix with all entries equal to 1, with $\sigma_{b}^{2}$ being the inter-individual variance. The intra-individual variability denoted by the 1xT vector $w_{s}$ is taken to be multivariate normally distributed with zero mean and covariance $D_{w}$. $D_{w}$ is a first-order autoregression covariance matrix of dimension T×T with entries being $D_{w}\left(i,j\right)=\sigma_{w}^{2}\rho^{\left|i-j\right|}$, where $\sigma_{w}^{2}$ is the intra-individual variance and ρ is the autocorrelation coefficient between two consecutive time points. The noise $\varepsilon_{s}$ is normally distributed with zero mean and covariance matrix $\sigma_{\varepsilon }^{2}1_{T}$, of dimension T×T.

Sixteen datasets with different numbers of subjects, numbers of variables, inter-individual variability, intra-individual variability and number of time points were generated with the above simulation method. In each of the sixteen datasets, eighty discriminating variables were simulated. Table S1 provides an overview of the characteristics of all the datasets. 

In the simulated dataset, the variables with the temporal profiles, (**a**)–(**f**) in Figure 1, were considered as discriminating variables, which are the target of variable selection while avoiding selection of variables with profiles (**g**)–(**h**) in Figure 1. 

#### 2.4.2. Onion Intervention Data

This data is taken from a randomized controlled trial with a crossover design, where participants were assigned to either an onion consuming group or a control group. Untargeted UPLC-qTOF-MS was applied to measure the metabolites in urine samples at four time points (0, 2, 4, 24 h after intervention) for six subjects per group [18]. The resulting raw data consists of 48 samples.

#### 2.4.3. Coffee Intervention Data

This data is generated from a randomized controlled trial with a crossover design, where urine samples were collected at 0, 0.5, 1, and 2 h after intervention with coffee or control drink (water) from 11 subjects per group. A total of 88 samples were analysed with untargeted UPLC-qTOF-MS [18]. 

Both onion and coffee raw data were converted to NetCDF files using DataBridge (Waters, Manchester, UK) and analysed with MZmine 2.19 for data peak detection, alignment and quantification [19]. The preprocessed data were imported into MATLAB and feature reduction was applied to remove unreliable variables due to compounds with extreme retention times, variables not detected in more than 70% of the samples in each subgroup and variables with a coefficient of variation (CV) in pooled quality control samples higher than 0.7 [20]. The resulting onion and coffee data sets had dimensions (samples x variables) of 48 × 3209 and 88 × 2321, respectively. 

For onion and coffee intervention data, true discriminating variables are not known *a priori*. However, to enable evaluation of the variable selection performance on this real data, ‘truly’ discriminating variables were determined by two methods. First, visual inspection was applied to identify variables exhibiting profiles similar to (**a**)–(**f**) in Figure 1. Second, a t-test was applied at each timepoint and the variable flagged if at least one time point was significant with a nominal *p <* 0.05. Variables were considered discriminating if both methods indicated a difference, and were the object of variable selection procedures.

### 2.5. Workflow

**Figure 3:**
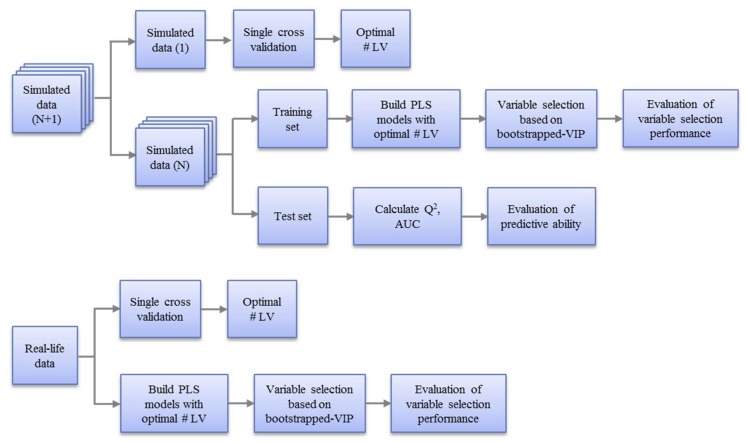
Workflow for the evaluation of variable selection performance of five PLS models on simulated datasets (top) and real datasets (bottom). For the simulated datasets, a single cross validation was applied on one of them to determine the optimal number of latent variables, which was then applied to all the similar simulated datasets for building the PLS models and variable selection. For real datasets, the optimal number of latent variables was obtained based on a single cross validation on the whole dataset and the PLS models were built on the whole dataset for variable selection.

The assessment of the variable selection of the five PLS models was performed on the simulated datasets as well as real datasets. The workflow is outlined in Figure 3 and explained in the following sections.

#### 2.5.1. Pre-Processing of Data

Centring and scaling are commonly applied prior to the regression modelling and have a critical influence on the performance of the model. Centring is performed to shift the mean of the data to zero and scaling is used to adjust the relative influence of variables with different variability. Centring across the first mode (samples or subjects) is a widely accepted step for both two-way and three-way data while scaling is more complicated. Scaling within one mode may disturb other modes [21,22]. In the current study, centring across the first mode was applied for both two-way and three-way data. For the two-way data, the values for each variable (column) were scaled to unit variance. On the three-way data, single-slab scaling within the metabolite mode was applied, as recommended by Gurden et al. [23]. (A slab is a single layer of the three-way array, here corresponding to a single variable). In single-slab scaling, each variable in the jth slab is scaled to unit root-mean-square of the slab (RMS_j_):

$${\mathrm{RMS}}_{j}=\sqrt{\frac{\sum_{s=1}^{S}\sum_{t=1}^{T}x_{\mathrm{sjt}}^{2}}{\mathrm{ST}}}$$

$$x_{\mathrm{sjt}}^{*}=\frac{x_{\mathrm{sjt}}}{{\mathrm{RMS}}_{j}}$$

where $x_{\mathrm{sjt}}$ is the intensity of metabolite j in the sample acquired from subject s at time point t, $x_{\mathrm{sjt}}^{*}$ is the single-slab scaled data.

#### 2.5.2. Model Optimization and Evaluation

For both simulated and real data, a single cross validation scheme was implemented, and the optimal number of latent variables was decided as the smallest number at which the decrease in root mean squared error in cross validation (RMSECV) between consecutive models was less than 2%. Due to the similarity of the repeated simulations using the same parameters, for the same type of PLS model, the number of latent variables was determined on one dataset and adopted for all the other repeats. 

A two-stage procedure was used to evaluate the performance of different models on simulated datasets. Each simulated dataset was divided into training and test sets. First, variable selection performance was evaluated on the training set. Next, the model’s predictive ability was evaluated on the test set.

##### (1). Evaluation of Variable Selection Performance with Training Sets

Balanced bootstrapping was performed to resample B bootstrap datasets [24,25]. Various values of B were tested and B = 200 was chosen as the smallest value providing consistent results (data not shown). PLS models with an optimal number of latent variables were built on each bootstrap subset and the Variable Importance in Projection (VIP) was calculated for each variable [26,27]. For each variable, the mean (VIP*) and standard deviation ($\sigma_{VIP}$) of the B VIP values were obtained. The variable was selected if the lower-bound of the one standard deviation error bar was above 1 (i.e., $VIP^{*}-\sigma_{VIP}>1$). 

To evaluate the variable selection performance of the five models, “Variable Selection ROC curves” were created. Since the discriminating variables are known, the model selecting the higher number of discriminating variables and lower number of non-discriminating variables is considered to have better variable selection performance. After the selected variables were obtained for each model, the number of variables considered as true positives, false positives, true negatives and false negatives were calculated according to Table 1. The comparison between convention ROC curve and Variable Selection ROC curve are shown in Figure S2. 

The area under the variable selection ROC curve (AUVSC) was calculated to provide an evaluation of the overall variable selection performance of each model. The following scores were calculated:

Recall = TP/(TP + FN)
Precision = TP/(TP + FP)
F_1_-score = (β^2^+1)× Precision × Recall/β^2^× (Precision + Recall)

Recall reflects the model’s capacity to select all the discriminating variables. Precision expresses the ability of the model to avoid the selection of non-discriminating variables. The F_1_-score is an overall assessment of the model’s performance on recall and precision, assessing the effectiveness of the model to identify all the discriminating variables without selecting too many non-discriminating variables. β is set to 1 to emphasize the importance of both recall and precision for a reasonable selection of variables.

##### (2). Evaluation of predictive ability with test sets

The models with the optimal number of latent variables determined on the training sets were applied (using all variables) to the corresponding test sets. Predictive variance explained Q^2^, and area under the conventional ROC curve (AUC, using all variables) were calculated to evaluate the predictive ability of the model.

For real datasets, stage (1) evaluation of variable selection performance was performed on the whole dataset. Stage (2) evaluation was not applied because the numbers of subjects are too small in the real datasets to obtain an independent test set. Instead, a permutation test was performed to evaluate the validity of the model. 

### 2.6. Evaluation of the Influence of Characteristics of the Dataset on the Model Performance 

Characteristics of metabolome vary, e.g., between different human studies, from humans to animals, and from studies on diets or drugs thereby leading to different characteristics of the datasets potentially influencing the performance of the statistical methods. To evaluate such influences and to provide guidelines for the use of different models, all five PLS models were applied on all sixteen simulated datasets as shown in Table S1. The results from different datasets were compared to assess the influence of characteristics on the model performance. The datasets used to compare the evaluation of different characteristics are shown in Table S2.

All the calculations were performed in MATLAB Version R2015b (8.6.0.267246) (The Mathworks, Inc, Natick, MA, USA) using scripts modified from N-way toolbox [28] and multiway VIP package [27]. The code for building the five PLS models and performing variable selection is available at https://github.com/qian-gao/PLSvar_sel. Simulated dataset 3 and anonymised onion intervention data are provided as examples for testing.

## 3. Results

### 3.1. Assessment of Variable Selection Performance on Simulated Data

#### 3.1.1. Overall Evaluation 

**Figure 4:**
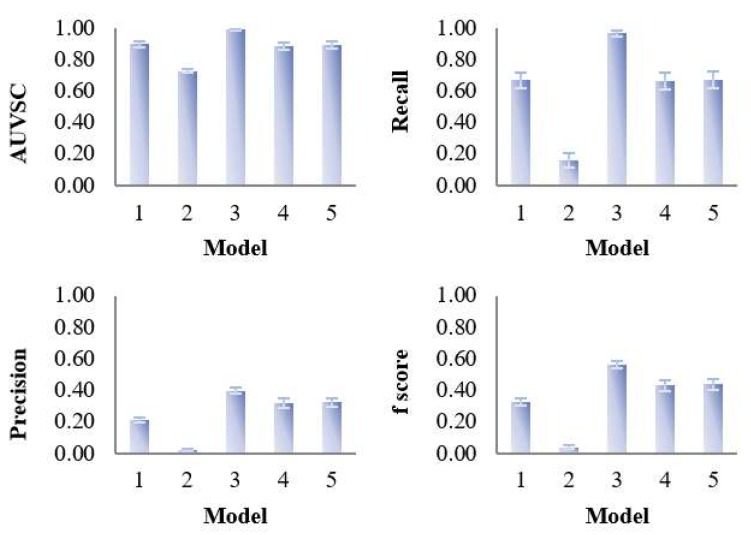
Evaluation of the variable selection performance of five PLS models on 100 simulated datasets. The variable selection performance consists of four criteria—area under the ROC curve (AUVSC), recall, precision, F_1_- score, which were calculated based on the variable selection confusion matrix.

The overall evaluation of the variable selection, prediction and classification performance of the five PLS models was performed on Dataset 3 (10 subjects, 3000 variables, 4 time points) and the results are shown in Table 2 and Figure 4. Dataset 3 was chosen for the overall evaluation because it has the characteristics that are most similar to those of the real dataset. As expected, bi-PLS models resulted in a higher number of latent variables than tri-PLS models indicating higher model complexity. Model 3 showed the best variable selection performance in that it provides the highest number of true positives with a relatively small number of selected variables, consequently leading to the highest precision. Model 1, 4 and 5 selected similar numbers of true positives while model 1 selected a higher number of false positives showing low precision. Model 2 showed the best prediction with highest Q^2^ but, as expected, provided a poor classification of the samples according to group. Unsurprisingly, only a few true positive variables were selected together with a large number of false positives resulting in the poorest precision. The number of latent variables did not have a strong influence on performance. Restricting all models to two latent variables (see Table S3), showed that the performance was not markedly different from that of those presented in Table 2.

**Figure 5:**
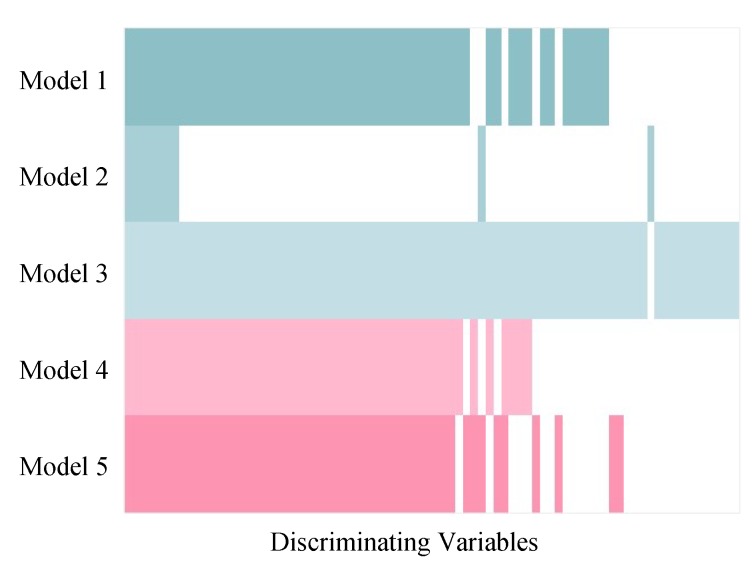
Comparison among discriminating variables selected by five PLS models in simulated Dataset 3. The coloured and white strips represent true positives (selected discriminating variables) and false negatives (unselected discriminating variables), respectively. The discriminating variables were arranged in order so that the variables selected by all five models were on the left side and the variables selected only by one model were on the right side.

**Figure 6:**
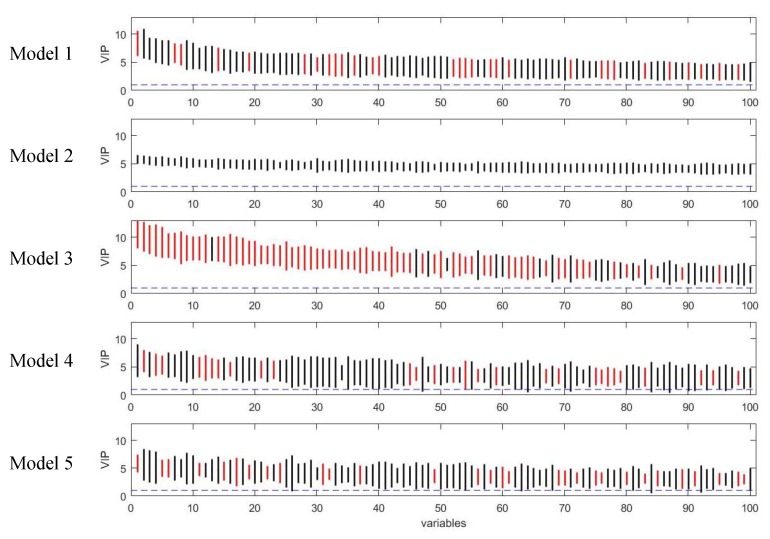
Rank of VIP scores for the discriminating variables in five PLS models on simulated Dataset 3. Bootstrapped VIP scores for all the variables were ranked according to their mean VIP scores in descending order. Bars show the mean +/- one standard deviation. Red and black represent the variables which are discriminating or non-discriminating, respectively. The horizontal blue dash line corresponds to VIP = 1.

Although the variable selection performances of the five models vary, the majority of discriminating variables were selected by at least two models, and variables selected by model 3 included approximate all the variables selected by other models (Figure 5). Beyond that, model 3 selected about eight unique true positives which were selected by none of the other models. The discriminating variables also had higher ranks in model 3 than the other models indicating its efficiency in variable selection (Figure 6). Model 4 and 5 resulted in low overall level of VIP scores and relatively larger variation, which caused a higher number of false negatives. 

#### 3.1.2. Influence of Characteristics of the Dataset on the Performance of the Five PLS Models

**Figure 7:**
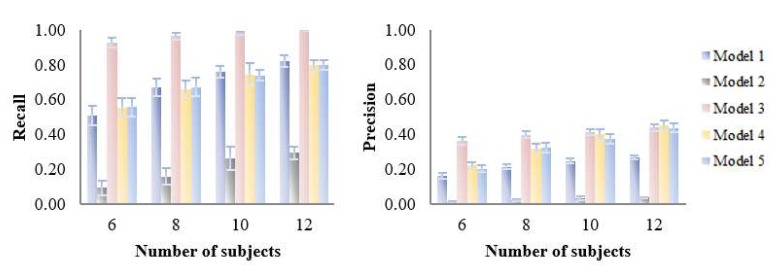
Influence of number of subjects (6–12) on the variable selection performance of five PLS models on simulated datasets. Recall and precision were calculated based on the variable selection confusion matrix.

The influence of number of subjects, number of variables, inter-individual variability, intra-individual variability and number of time points was assessed with the simulated datasets and the results are shown in Figure 7, Table 3, Table 4, Figure S3 and Figure S4.

As expected, variable selection performance (recall and precision) of all models was improved with increasing number of subjects (Figure 7). Notably, model 3 was only slightly affected by the number of subjects as it maintained its good recall and precision throughout all datasets, suggesting good robustness to this parameter.

Not surprisingly, the increased number of noisy variables in the data led to a higher number of selected variables and most of the extra selected variables are false positives (Table 3). The number of true positives in model 1–3 was not affected by the noisy variables while Model 4 and 5 selected fewer true positives but also fewer false positives under the influence of noise.

Variable selection performances of the five models were strongly affected by inter-individual variability in that all the models except model 2 selected fewer true positives with larger inter-individual variability (Table 4). When the inter-individual variability increased, bi-PLS models tended to maintain their recall by sacrificing the precision; tri-PLS models tended to maintain the precision by keeping a stable number of selected variables. Overall, model 3 was less affected by inter-individual variability than other models showing a good trade-off between recall and precision. 

Intra-individual variability had little influence on the variable selection performance of five PLS models (Figure S3). As expected, a higher number of time points led to better recall of all the five models and model 1 benefited most from the extra temporal information (Figure S4).

### 3.2. Assessment of Variable Selection Performance on Real Data

**Figure 8:**
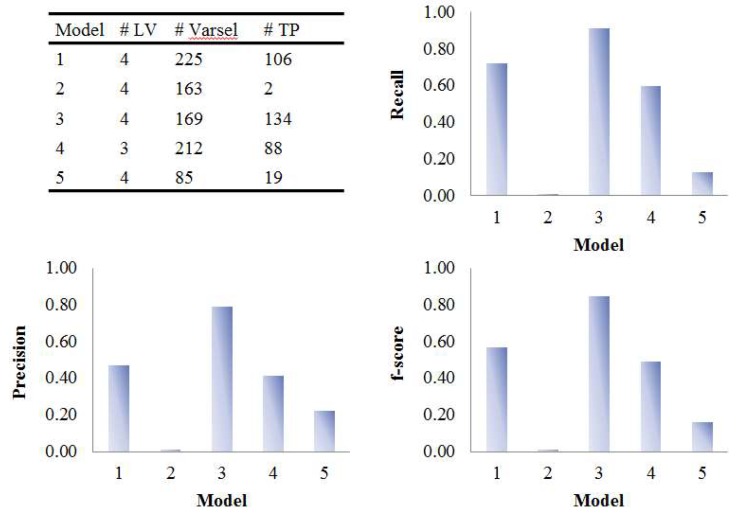
Evaluation of the variable selection performance of five PLS models on onion study data. Recall, precision and F_1_-score were calculated based on the variable selection confusion matrix. # LV, number of latent variables; # Varsel, number of selected variables; # TP, number of true positives.

**Figure 9:**
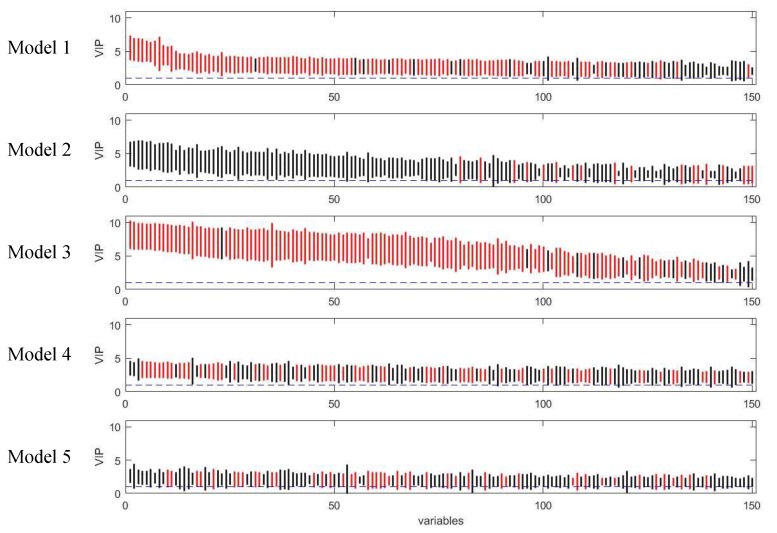
Rank of VIP scores for the discriminating variables in five PLS models on onion study dataset. Bootstrapped VIP scores for all the variables were ranked according to their mean VIP scores in descending order. Red and black represent the variables which are discriminating or non-discriminating, respectively. The horizontal blue dash line corresponds to VIP = 1.

The five PLS models were applied on the onion intervention data to discover variables discriminating the control and intervention groups and the results are shown in Figure 8 and Figure 9. Similar to the simulated dataset, Model 3 provided the best recall and precision resulting in around 28 more true positives than the second best, model 1. Again, most of the discriminating variables had high ranks in model 3. Model 4 and model 5 were not capable of selecting many true positives in this more challenging real dataset, perhaps due to their tendency to maintain precision by keeping a low number of selected variables when dealing with data having large inter-individual variability. The low overall level of VIP scores and relatively large variation could also be the reason why so few variables were selected in Model 5. Interestingly, when variables were selected according to loading weights (instead of VIP), the performance of model 4 and 5 was improved, and was similar to the performance of model 1, but still not better than Model 3 (see Figure S6). A permutation test was performed which showed that Model 3 was significant at *p <* 0.001 (Figure S9).

#### 3.2.1. Coffee Intervention Study

**Figure 10:**
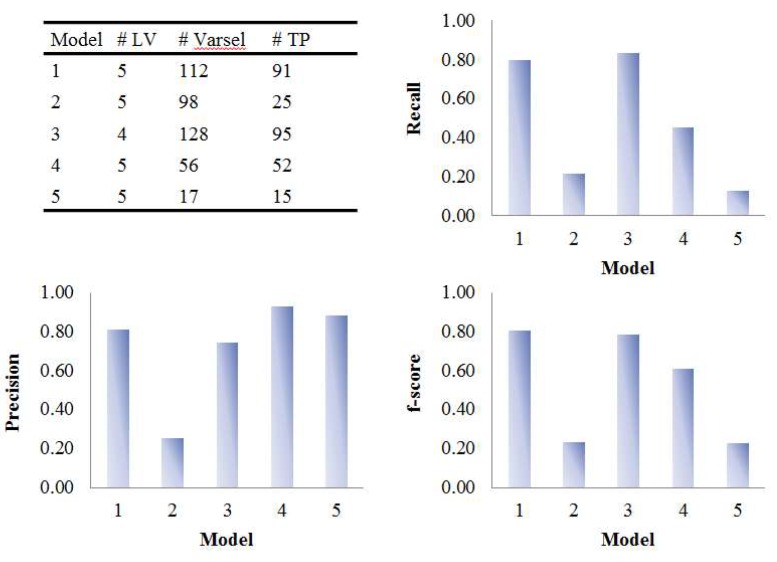
Evaluation of the variable selection performance of five PLS models on coffee study data. Recall, precision and F_1_-score were calculated based on the variable selection confusion matrix. # LV, number of latent variables; # Varsel, number of selected variables; # TP, number of true positives.

**Figure 11:**
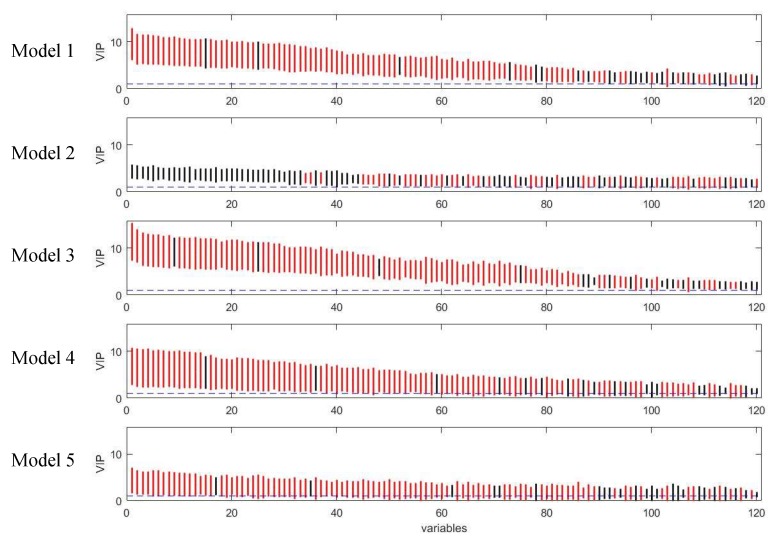
Rank of VIP scores for the discriminating variables in five PLS models on coffee study dataset. Bootstrapped VIP scores for all the variables were ranked according to their mean VIP scores in descending order. Red and black represent the variables which are discriminating or non-discriminating, respectively. The horizontal blue dash line corresponds to VIP = 1.

In coffee intervention study, urine samples were collected at 0, 0.5, 1, and 2 h after intervention. Due to the short sample collection period, the temporal profiles of metabolites were incomplete as shown in Figure S7. In this case, the time response class was labelled as 1 for the samples collected at 0 h and 10 for the samples collected at 0.5, 1, and 2 h after intervention. The performances of the five PLS models on coffee intervention data were similar to that for simulated data and the results were shown in Figure 10 and Figure 11. Model 3 gave the highest number of true positives with a reasonable number of selected variables. It also provided the most comprehensive list of selected variables; its selection of true positives included almost all the true positives found in all the other models (see Figure S8). The permutation test (Figure S9) indicated Model 3 was significant at *p <* 0.001 confirming that it was not overfitted and therefore its good variable selection performance was valid. Tri-PLS models were very conservative in that they selected fewer variables but gave very high precision. In fact, the discriminating variables had better ranks in tri-PLS models than in Model 1, so that if we lower the threshold for bootstrapped-VIP scores, model 4 and 5 would outperform model 1.

## 4. Discussion

In this paper, five PLS modelling approaches for metabolomic time series data were evaluated with simulated and real data with the objective of identifying variables showing discriminatory temporal patterns. The variable selection performance of the models was compared on simulated datasets based on their capacity to select discriminating variables while avoiding non-discriminating variables. The influence on model performance of five factors (number of subjects, number of variables, inter-individual variability, intra-individual variability and number of time points) was assessed to provide additional information on the application of suitable models for different scenarios of data. 

Several issues have been considered regarding the development of these models. Bootstrapped-VIP scores were calculated to evaluate the importance of variables in the current paper. This approach was shown in previous studies [4,29,30] to be sensitive and precise in selecting relevant variables. However, we are aware that it might not always be the optimal approach for all models or datasets. For example, in the analysis of the onion intervention study, the loading weight for the first component was a more powerful selection tool for N-PLS models. This might be due to the fact that loading weight for the first component directly reflects the covariance between **X** and **Y**. Additional components that are found in the residuals after removal of previous components from **X** might be influenced by irrelevant information in **X** [31]. Therefore, the inclusion of the information from extra components does not necessarily result in better variable selection performance of the model. This may also explain why no strong difference in variable selection performance was observed between the models with different number of latent variables. 

Another issue is the applicability of the proposed model to data with incomplete temporal profiles, where metabolite levels may not return to pre-intervention levels. For example, the coffee intervention study collected data at 0 h and 0.5, 1, and 2 h after intervention which means the whole excretion profile of the metabolites might not be recorded since the sampling period was too short (Figure S7). Our results from the coffee study indicate that incomplete temporal profiles can still provide information for the identification of discriminating variables as long as the ‘response class’ and ‘non-response class’ (i.e., responding and nonresponding time points) are accurately assigned. 

The resulting models were assessed on both simulated and real data and our results were consistent in showing that 1) The bi-PLS model with combined time response and group information as **Y** (model 3) had the best variable selection performance and the most comprehensive list of true positives for all datasets tested. 2) The tri-PLS models tested both tend to maintain high precision by sacrificing recall, however they show robust performance on data with a high number of noise variables. 3) In datasets with high inter-individual variability, bi-PLS models tend to provide higher recall while tri-PLS models tend to provide higher precision. As expected, the bi-PLS model with time response as **Y** (model 2) performed most poorly under all conditions confirming that time response alone is not enough to discriminate samples from different classes.

Discovery, identification and validation of biomarkers in metabolomic studies is difficult and time-consuming. The goal is often to provide a list of discriminating variables with as many true positives and as few false positives as possible. Based on this goal and our comparison between the five models, Model 3 provided both good recall and precision and therefore represents a good choice for suitable datasets with time response profiles in two treatment groups. When the time dependant response is not recorded, model 1 and 4 may be adopted as the best general approach and they can be selected in different situation depending on the purpose of the study. For instance, Model 1 would be a good choice for exploring the data and collecting as many relevant variables as possible since it tends to keep high recall at any costs. For studies aiming at finding biomarkers with potential to classify new samples, model 4 has the potential to select the most suitable metabolites because of its good precision. 

Time-series designs are widely used in life science research and the purpose is to observe the response of a biological system to a certain challenge over a defined time period. Although this work is demonstrated with LC-MS metabolomic data, it is applicable also to other types of multivariate time-series data, such as RNA-seq experiments aiming to detect the gene expression differences between experimental groups. Several methods have been proposed previously to deal with this type of time-series data. Bar-Joseph et al. [32] describes gene expression over time as a continuous curve and identifies genes showing significant temporal expression differences based on the difference between the curves. To accurately fit the curve representing the temporal profile, this method usually requires relatively long time series and homogeneous data which is not often available due to limitations of the study design or high inter-individual variability. Compared to this method, in our study Model 3 successfully dealt with short time series data and maintained high recall and precision even in the presence of high inter-individual variability. Regression-based methods have also been developed where gene expression is described as a function of time and regression coefficients of each gene from different experimental groups are compared using ANOVA [33]. Compared to this method, our Model 3 and 5 retain the multivariate structure and thus take correlations between variables into account. ANOVA-simultaneous component analysis (ASCA) is another popular method that can be applied to time-series data [34]. The data is separated into the variations that contributed by different experimental design factors such as time, dose of intervention, and their interactions using ANOVA equation. Simultaneous component analysis is then applied to different variations to approximate the scores and loadings in each sub-model. ASCA is efficient in separating design factors and exploring the data correspondingly. However, it is not able to select variables with specific response profiles (e.g., (a)–(f)) as our models do but only indicate if there is an overall difference. Moreover, these five models have low computational cost.

In summary, both simulated and real data demonstrate that bilinear PLS model with group × time response as dummy **Y** is a powerful method for variable selection in time-series experiments. It maintains good performance in the presence of noise and high inter-individual variability. In general, bi-PLS models tend to provide higher recall while tri-PLS models tend to provide higher precision.

## Figures and Tables

**Table 1:** Variable selection confusion matrix.

		True Condition	
	Total Variables	Discriminating Variables	Non-Discriminating Variables
Predicted Condition	Selected variables	True positive (TP)	False positive (FP)
	Unselected variables	False negative (FN)	True negative (TN)

**Table 2:** Performance of five PLS models evaluated on simulated datasets with the optimal number of latent variables.

Model	Data (X or X)	Dummy Y	# LV^a^	Predictive Ability		Variable Selection Performance	
				Q^2^	AUC	# Varsel^b^	# TP^c^
1	Mode 1: Sample Mode 2: Metabolite	Group	3	0.57 (0.02)	0.93 (0.06)	249.3 (11.2)	53.6 (4)
2		Time response	5	1 (0)	0.58 (0.08)	591.1 (16)	12.8 (3.9)
3		Group × Time response	5	0.83 (0.01)	0.75 (0)	194.5 (9.1)	77.3 (1.6)
4	Mode 1: Subject Mode 2: Metabolite Mode 3: Time	Group	1	0.6 (0.01)	1 (0)	165.9 (11.4)	53 (4.1)
5		Mode 1: Group Mode 3: Time	1	0.6 (0.02)	0.75 (0)	166.7 (13.5)	53.9 (4.3)

^a^ # LV, number of latent variables; ^b^ # Varsel, number of selected variables; ^c^ # TP, number of true positives. Values reported are mean and standard deviation across 100 repeats.

**Table 3:** Influence of the number of variables on variable selection performance of the five models on simulated data.

Model.	Number of Variables (No. Discriminating Variables Kept at 80 in All Cases).							
	1000		3000		5000		7000	
	# Varsel^a^	# TP^b^	# Varsel	# TP	# Varsel	# TP	# Varsel	# TP
1	105 (5.2)	51.7 (3.1)	249.3 (11.2)	53.6 (4)	396.3 (12.2)	53.9 (4)	540.4 (20.3)	53.3 (4.4)
2	189.8 (6.9)	15.1 (3.9)	591.1 (16)	12.8 (3.9)	983.1 (19.8)	15.8 (3.7)	1379.1 (22.1)	13.7 (2.1)
3	95.7 (4.6)	74.3 (1.8)	194.5 (9.1)	77.3 (1.6)	304.7 (14.8)	77.1 (2.2)	409.3 (18.2)	77.5 (1.4)
4	86.2 (7.1)	58 (3.6)	165.9 (11.4)	53 (4.1)	243.8 (18.8)	50.2 (4.1)	325.8 (21.7)	49.6 (2.9)
5	89 (5.2)	58.4 (3.8)	166.7 (13.5)	53.9 (4.3)	247.4 (14.8)	51.7 (2.8)	325.2 (15.6)	51.3 (3.3)

^a^ # Varsel, number of selected variables; ^b^ # TP, number of true positives. Values reported are mean and standard deviation across 100 repeats.

**Table 4:** Influence of inter-individual variability on variable selection performance of five models on simulated data.

Model	Inter-Individual Variability							
	0.1		0.3		0.5		0.7	
	# Varsel^a^	# TP^b^	# Varsel	# TP	# Varsel	# TP	# Varsel	# TP
1	153.5 (8.2)	75.1 (1.9)	249.3 (11.2)	53.6 (4)	301 (11.5)	39.2 (3.8)	323 (11.1)	34.2 (3.3)
2	681.5 (17.6)	2.7 (1.4)	591.1 (16)	12.8 (3.9)	513.5 (15.3)	19.4 (3)	493 (18.3)	19.2 (3.6)
3	143.5 (4.5)	79.9 (0.3)	194.5 (9.1)	77.3 (1.6)	216.5 (14.4)	72.2 (2.5)	243.1 (13.3)	68.3 (3.5)
4	173.9 (12.1)	78.7 (1.2)	165.9 (11.4)	53 (4.1)	155.2 (11.6)	35.9 (4.3)	165.9 (15.8)	27 (2.5)
5	177.5 (12.5)	79.2 (0.9)	166.7 (13.5)	53.9 (4.3)	158.8 (12.7)	35.4 (3.4)	170.1 (13.4)	28.1 (5)

^a^ # Varsel, number of selected variables; ^b^ # TP: number of true positives. Values reported are mean and standard deviation across 100 repeats.

## Supplementary Materials

The following are available online at https://www.mdpi.com/2218-1989/9/5/92/s1. Figure S1: Temporal profiles of metabolites in simulated data with a time-series design. Figure S2: Convention ROC curve and Variable Selection ROC curve (VSROC) for a. model 1 and b. model 3 in simulated Dataset 3. Figure S3: Influence of intra-individual variability (0.1–0.4) on the variable selection performance of five PLS models on simulated datasets. Figure S4: Influence of number of time points (3–6) on the variable selection performance of five PLS models on simulated datasets. Figure S5: Comparison among discriminating variables selected by five PLS models in onion study data. Figure S6: Comparison between the variable selection performances based on loading weight (green) and VIP (blue) of five PLS models on onion study data. Figure S7: Temporal profiles of metabolites observed in our coffee data with a time-series design. Figure S8: Comparison among discriminating variables selected by five PLS models in coffee study data. Figure S9: Area under the ROC curve (AUC) calculated on permuted **Y** data for model 3 (histogram, 1000 permutations) and original data (red dot) generated on samples obtained from onion (left) and coffee (right) studies. Table S1: Characteristics of sixteen simulated datasets. Table S2: Datasets used to compare for the evaluation of different factors. Table S3: Performance of five PLS models evaluated on simulated dataset with same number of latent variables. Table S4: Parameters used for pre-processing the onion study data in MZmine2. Table S5: Parameters used for pre-processing the coffee study data in MZmine2.

## References

[1] Rezzi S., Ramadan Z., Fay L.B., Kochhar S. (2007). Nutritional metabonomics: applications and perspectives. J. Proteome Res..

[2] Broadhurst D.I., Kella D.B. (2008). Statistical strategies for avoiding false discoveries in metabolomics and related experiments. Metabolomics.

[3] Brennan L. (2013). Metabolomics in nutrition research: current status and perspectives. Biochem. Soc. Trans..

[4] Christin C., Hoefsloot H.C.J., Smilde A.K., Hoekman B., Suits F., Bischoff R., Horvatovich P. (2013). A critical assessment of feature selection methods for biomarker discovery in clinical proteomics. Mol. Cell. Proteom..

[5] Dragsted L.O., Gao Q., Scalbert A., Vergères G., Kolehmainen M., Manach C., Brennan L., Afman L.A., Wishart D.S., Andres-Lacueva C., Garcia-Aloy M., Verhagen H., Feskens E.J.M., Praticò G. (2018). Validation of biomarkers of food intake: critical assessment of candidate biomarkers. Genes Nutr..

[6] Szymańska E., Saccenti E., Smilde A.K., Westerhuis J.A. (2012). Double-check: validation of diagnostic statistics for PLS-DA models in metabolomics studies. Metabolomics.

[7] Barker M., Rayens W. (2003). Partial least squares for discrimination. J. Chemom..

[8] Wold S., Kettaneh N., Fridén H., Holmberg A. (1998). Modelling and diagnostics of batch processes and analogous kinetic experiments. Chemom. Intell. Lab. Syst..

[9] Antti H., Bollard M.E., Ebbels T., Keun H., Lindon J.C., Nicholson J.K., Holmes E. (2002). Batch statistical processing of1H NMR-derived urinary spectral data. J. Chemom..

[10] Jonsson P., Stenlund H., Moritz T., Trygg J., Sjöström M., Verheij E.R., Lindberg J., Schuppe-Koistinen I., Antti H. (2006). A strategy for modelling dynamic responses in metabolic samples characterized by GC/MS. Metabolomics.

[11] Rantalainen M., Cloarec O., Ebbels T.M.D., Lundstedt T., Nicholson J.K., Holmes E., Trygg J. (2008). Piecewise multivariate modelling of sequential metabolic profiling data. BMC Bioinform..

[12] Kusalik A.J. (2004). State-space model with time delays for gene regulatory networks. J. Biol. Syst..

[13] Smilde A.K., Westerhuis J.A., Hoefsloot H.C.J., Bijlsma S., Rubingh C.M., Vis D.J., Jellema R.H., Pijl H., Roelfsema F., van der Greef J. (2010). Dynamic metabolomic data analysis: a tutorial review. Metabolomics.

[14] Wold S., Sjöström M., Eriksson L. (2001). PLS-regression: a basic tool of chemometrics. Chemom. Intell. Lab. Syst..

[15] Bro R. (1996). Multiway calibration. Multilinear PLS. J. Chemom..

[16] Rubingh C.M., Bijlsma S., Jellema R.H., Overkamp K.M., Van Der Werf M.J., Smilde A.K. (2009). Analyzing longitudinal microbial metabolomics data. J. Proteome Res..

[17] Andersen C.M., Bro R. (2010). Variable selection in regression-a tutorial. J. Chemom..

[18] Barri T., Holmer-Jensen J., Hermansen K., Dragsted L.O. (2012). Metabolic fingerprinting of high-fat plasma samples processed by centrifugation-and filtration-based protein precipitation delineates significant differences in metabolite information coverage. Anal. Chim. Acta.

[19] Gürdeniz G., Kristensen M., Skov T., Dragsted L.O. (2012). The effect of LC-MS data preprocessing methods on the selection of plasma biomarkers in fed vs. *fasted rats*. Metabolites.

[20] Gürdeniz G., Jensen M.G., Meier S., Bech L., Lund E., Dragsted L.O. (2016). Detecting beer intake by unique metabolite patterns. J. Proteome Res..

[21] Smilde A., Bro R., Geladi P. (2005). Multi-way Analysis: Applications in the Chemical Sciences.

[22] Kiers H.A.L., Van Mechelen I. (2001). Three-way component analysis: Principles and illustrative application. Psychol. Methods.

[23] Gurden S.P., Westerhuis J.A., Bro R., Smilde A.K. (2001). A comparison of multiway regression and scaling methods. Chemom. Intell. Lab. Syst..

[24] Gosselin R., Rodrigue D., Duchesne C. (2010). A Bootstrap-VIP approach for selecting wavelength intervals in spectral imaging applications. Chemom. Intell. Lab. Syst..

[25] Gleason J.R. (1988). Algorithms for balanced bootstrap simulations. Am. Stat..

[26] Wold S., Johansson E., Cocchi M. (1993). 3D QSAR in Drug Design: Theory, Methods and Applications.

[27] Favilla S., Durante C., Vigni M.L., Cocchi M. (2013). Assessing feature relevance in NPLS models by VIP. Chemom. Intell. Lab. Syst..

[28] Andersson C.A., Bro R. (2000). The N-way Toolbox for MATLAB. Chemom. Intell. Lab. Syst..

[29] Chong I.-G., Jun C.-H. (2005). Performance of some variable selection methods when multicollinearity is present. Chemom. Intell. Lab. Syst..

[30] Alves A.C., Li J.V., Garcia-Perez I., Sands C., Barbas C., Holmes E., Ebbels T.M.D. (2012). Characterization of data analysis methods for information recovery from metabolic 1H NMR spectra using artificial complex mixtures. Metabolomics.

[31] Gidskehaug L., Anderssen E., Flatberg A., Alsberg B.K. (2007). A framework for significance analysis of gene expression data using dimension reduction methods. BMC Bioinform..

[32] Bar-Joseph Z., Gerber G., Simon I., Gifford D.K., Jaakkola T.S. (2003). Comparing the continuous representation of time-series expression profiles to identify differentially expressed genes. Proc. Natl. Acad. Sci. USA.

[33] Berk M., Ebbels T., Montana G. (2011). A statistical framework for biomarker discovery in metabolomic time course data. Bioinformatics.

[34] Smilde A.K., Jansen J.J., Hoefsloot H.C.J., Lamers R.J.A.N., van der Greef J., Timmerman M.E. (2005). ANOVA-simultaneous component analysis (ASCA): A new tool for analyzing designed metabolomics data. Bioinformatics.

