# Supplementary material for: Comparison of Bi- and Tri-Linear PLS Models for Variable Selection in Metabolomic Time-Series Experiments
Source: Metabolites. 2019 May 9;9(5):92. doi: 10.3390/metabo9050092 (PMC6571821; doi:10.3390/metabo9050092)
Supplement: Supplementary file 1 [file metabolites-09-00092-s001.pdf]

Comparison of bi- and tri-linear PLS models for variable selection in metabolomic time series experiments

Qian Gao [1], Lars O. Dragsted [1] and Timothy Ebbels [2,*]

[1] Department of Nutrition, Exercise and Sports, University of Copenhagen, Copenhagen, Denmark; qian@nexs.ku.dk (Q.G.); ldra@ nexs.ku.dk (L.O.D.)

[2] Computational and Systems Medicine, Department of Surgery and Cancer, Imperial College London, London, United Kingdom; t.ebbels@imperial.ac.uk

* Correspondence: t.ebbels@imperial.ac.uk; Tel.: +44 20 75 94 31 60

Supplementary material

Text S1. In acute intervention metabolomic studies (< 24 h), metabolites respond differently between the groups and typically show eight different types of temporal profiles: (a) intervention positive response, control no response, (b) intervention no response, control negative response, (c) intervention no response, control positive response, (d) intervention negative response, control no response, (e) intervention positive response, control negative response, (f) intervention negative response, control positive response, (g) intervention and control the same response, (h) intervention and control random response.

Table S1. Characteristics of sixteen simulated datasets.

Factors	Datasets															
	1	2	3	4	5	6	7	8	9	10	11	12	13	14	15	16
Number of subjects	6	8	10	12	8	8	8	8	8	8	8	8	8	8	8	8
Number of variables	3000	3000	3000	3000	3000	3000	3000	3000	3000	3000	3000	3000	3000	1000	5000	7000
Inter-individual variability	0.3	0.3	0.3	0.3	0.3	0.3	0.3	0.1	0.5	0.7	0.3	0.3	0.3	0.3	0.3	0.3
Intra-individual variability	0.2	0.2	0.2	0.2	0.2	0.2	0.2	0.2	0.2	0.2	0.1	0.3	0.4	0.2	0.2	0.2
Number of time points	4	4	4	4	3	5	6	4	4	4	4	4	4	4	4	4

Table S2. Datasets used to compare for the evaluation of different factors.

Factor to evaluate	Datasets to compare
Number of subjects	1, 2, 3, 4
Number of variables	2, 14, 15, 16
Inter-individual variability	2, 8, 9, 10
Intra-individual variability	2, 11, 12, 13
Number of time points	2, 5, 6, 7

Table S3. Performance of five PLS models evaluated on simulated dataset with same number of latent variables.

Model	# LV	Q2	AUC	AUVSC	# Varsel[a]	# TP[b]
1	2	0.58 (0.02)	0.86 (0.05)	0.89 (0.02)	249.1 (9.1)	55.2 (4.7)
2	2	1 (0)	0.6 (0.08)	0.72 (0.01)	607.8 (10.9)	14.3 (4.7)
3	2	0.77 (0.02)	0.74 (0.02)	0.99 (0)	192.7 (10)	76 (1.4)
4	2	0.6 (0.01)	1 (0)	0.88 (0.02)	171.4 (11.3)	52.4 (3.6)
5	2	0.6 (0.01)	0.75 (0)	0.87 (0.02)	174.3 (11)	52.2 (4.7)

[a] # LV, number of latent variables; [b] # Varsel, number of selected variables; [c] # TP, number of true positives.

Table S4. Parameters used for pre-processing the onion study data in MZmine2.

Batch step	Parameters
Raw data import	
Mass detection	Noise level: 15
Chromatogram builder	Min time span (min): 0.02;<br>Min height: 5.0E1; m/z tolerance: 0.04 mz or 30 ppm
Chromatogram deconvolution	Chromatographic threshold: 5%; Search minimum in RT range (min): 0.01; Minimum relative height: 5%; Minimum absolute height: 5.0E1; Min ratio of peak/top edge: 1.5; Peak duration range (min): 0.01-0.2
Isotopic pattern	m/z tolerance: 0.08 or 60 ppm; Retention time tolerance: 0.02; Monotonic shape; maximum charge: 1
Join aligner	m/z tolerance: 0.06 or 30 ppm; Absolute retention time tolerance: 0.06; Weight for both m/z tolerance and retention time tolerance: 10
Duplicate peak filter	m/z tolerance: 0.2 or 200 ppm; RT tolerance: 0.01

Peak list rows filter	Min peaks in a row: 5<br>Minimum peaks in an isotope pattern: 1; m/z range: 50-1000; RT range: 0-7;
Peak finder	Intensity tolerance: 1%; m/z tolerance: 0.04 or 30 ppm; Absolute retention time tolerance: 0.02

Table S5. Parameters used for pre-processing the coffee study data in MZmine2.

Batch step	Parameters
Raw data import	
Mass detection	Noise level: 20
Chromatogram builder	Min time span (min): 0.02;<br>Min height: 4.0E1; m/z tolerance: 0.04 mz or 30 ppm
Chromatogram deconvolution	Chromatographic threshold: 5%; Search minimum in RT range (min): 0.01; Minimum relative height: 5%; Minimum absolute height: 4.0E1; Min ratio of peak/top edge: 1.4; Peak duration range (min): 0.01-0.2
Isotopic pattern	m/z tolerance: 0.08 or 60 ppm; Retention time tolerance: 0.02; Monotonic shape; maximum charge: 1
Join aligner	m/z tolerance: 0.06 or 30 ppm; Absolute retention time tolerance: 0.06; Weight for both m/z tolerance and retention time tolerance: 10
Duplicate peak filter	m/z tolerance: 0.2 or 200 ppm; RT tolerance: 0.01
Peak list rows filter	Min peaks in a row: 7<br>Minimum peaks in an isotope pattern: 1; m/z range: 50-1000; RT range: 0-7;
Peak finder	Intensity tolerance: 1%; m/z tolerance: 0.04 or 30 ppm; Absolute retention time tolerance: 0.02

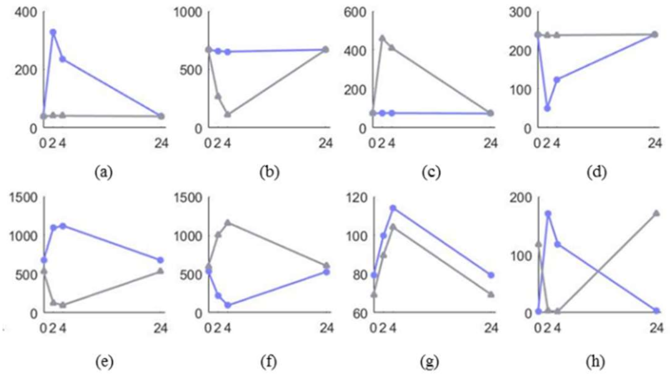

Figure S1. Temporal profiles of metabolites in simulated data with a time-series design. (a)-(h) are temporal profiles of eight metabolites simulated for two subject in control (grey) and intervention (purple) group, respectively. For each type of metabolites in each group, c, a, α, β are adjusted to create temporal profiles shown in Fig 1 (a)-(h) correspondingly. For instance, to simulate metabolite (a) in intervention group, the parameters are generated as follows: c~U(1,100), a~U(100,500), α ~U(5,20), β~U(40,50).

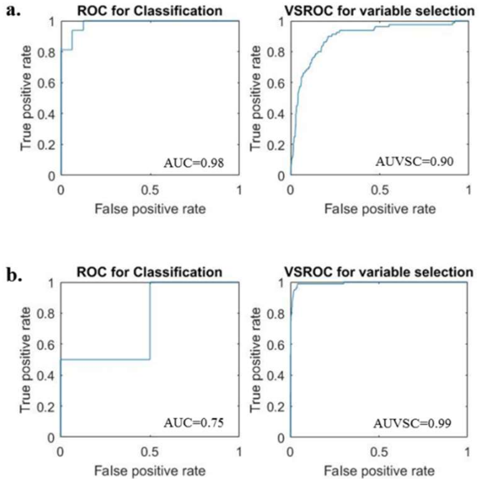

Figure S2. Convention ROC curve and Variable Selection ROC curve (VSROC) for **a.** model 1 and **b.** model 3 in simulated Dataset 3. Convention ROC and VSROC were used to evaluate the classification and variable selection performance of the model, respectively.

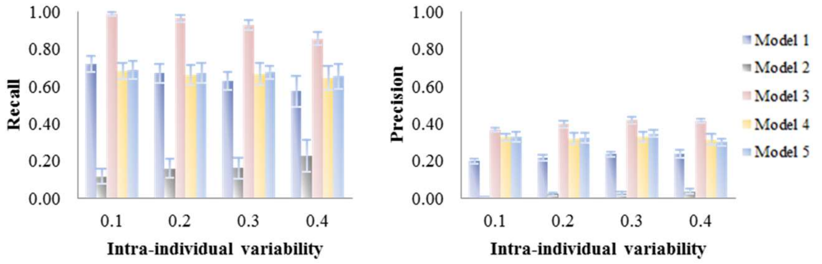

Figure S3. Influence of intra-individual variability (0.1-0.4) on the variable selection performance of five PLS models on simulated datasets. Recall and precision were calculated based on the variable selection ROC curve.

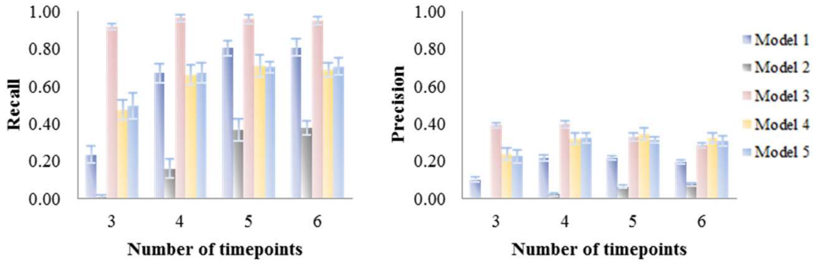

Figure S4. Influence of number of time points (3-6) on the variable selection performance of five PLS models on simulated datasets. Recall and precision were calculated based on the variable selection confusion matrix.

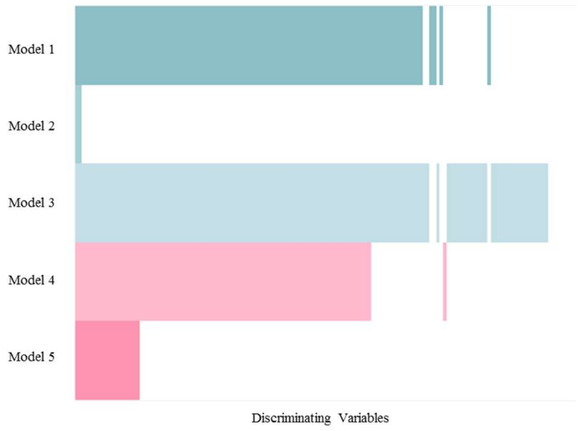

Figure S5. Comparison among discriminating variables selected by five PLS models in onion study data. The coloured and white strips represent true positives (selected discriminating variables) and false negatives (unselected discriminating variables), respectively. The discriminating variables were arranged in order so that the variables selected by all five models were on the left side and the variables selected only by one model were on the right side.

Model	# LV	#Varsel	# TP based on w	# TP based on VIP
1	4	150	90	108
2	4	150	10	18
3	4	150	128	131
4	3	150	88	66
5	4	150	88	47

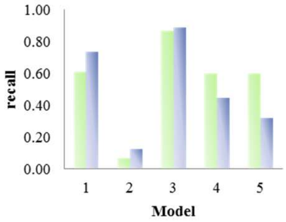

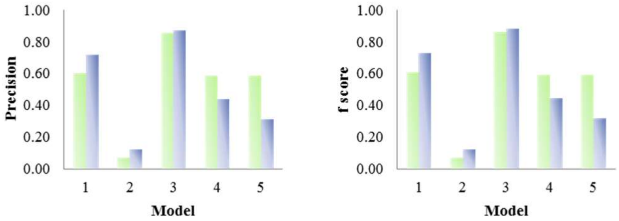

Figure S6. Comparison between the variable selection performances based on loading weight (green) and VIP (blue) of five PLS models on onion study data. recall, precision and f-score were calculated based on the variable selection confusion matrix. # LV, number of latent variables; # Varsel, number of selected variables; # TP based on w, true positives selected based on loading weights; # TP based on VIP, true positives selected based on VIP scores. Bootstrap procedure was not used here because when it applied to the variable selection based on loading weights, almost all the variables were selected with the threshold setting to 0 ($w^* - \sigma_w > 0$, w^* and σ_w are mean and standard deviation of the B w values). 150 variables with highest w or VIP were selected in each model.

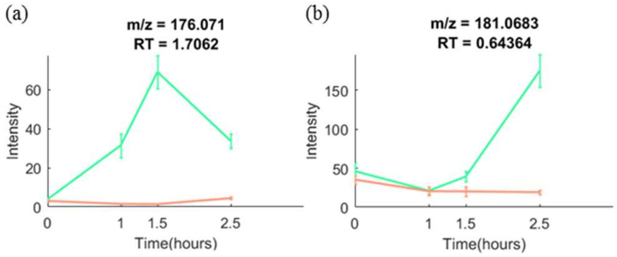

Figure S7. Temporal profiles of metabolites observed in our coffee data with a time-series design. (a)-(b) are temporal profiles of two metabolites in control (orange) and intervention (green) group, respectively.

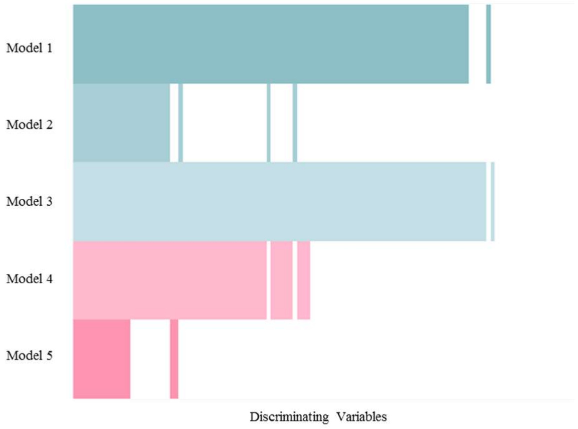

Figure S8. Comparison among discriminating variables selected by five PLS models in coffee study data. The coloured and white strips represent true positives (selected discriminating variables) and false negatives (unselected discriminating variables), respectively. The discriminating variables were arranged in order so that the variables selected by all five models were on the left side and the variables selected only by one model were on the right side.

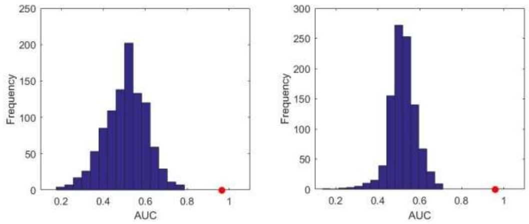

Figure S9. Area under the ROC curve (AUC) calculated on permuted Y data for model 3 (histogram, 1000 permutations) and original data (red dot) generated on samples obtained from onion (left) and coffee (right) studies. In both cases it is clear that the AUC obtained from the non-permuted Y is in excess of all values obtained through permutation, indicating that the models do not overfit and are significant at an empirical $p<0.001$.
